# The Elderly and the COVID 19 Crisis: A Chronicle of Deaths Foretold, in Isolation and Total Indifference

**DOI:** 10.3389/fpubh.2020.602982

**Published:** 2021-01-08

**Authors:** Dominique Heymann

**Affiliations:** ^1^Université de Nantes, Institut de Cancérologie de l'Ouest, Saint-Herblain, France; ^2^Department of Oncology and Metabolism, University of Sheffield, Sheffield, United Kingdom

**Keywords:** COVID-19 pandemic, elderly, health authorities, anxiety, depression disorders

## Introduction

Since December 2019, the COVID 19 pandemic has been an unprecedented health crisis that has shaken the entire planet, dragging the social, economic, and political world into turmoil. The elderly population is one of the prime targets of COVID 19, finding itself on the front line and, as a result, it has already paid a heavy price. In many countries, the elderly account for almost half of the deaths that have occurred in the past 3 months (1, 2).

## COVID-19 Crisis: The Elderly Face to the Public Health Authorities

The elderly are our collective memory, with their successes, mistakes, and failures, and they thus occupy a specific place in industrialized countries. As global populations age, the elderly have logically become more numerous, leading countries to rethink national organization of care infrastructures. This in turn has attracted the attention of numerous commercial interests. Faced with this unprecedented situation, the total closure of nursing homes and home care services for seniors as a means of respecting strict social distancing measures has been imposed by many governments, and recommendations have been defined for those who stayed at home (3). These decisions were justified as a way of protecting those who are the most vulnerable, and societies trusted their politicians.

Protecting the elderly was legitimized by the severity of this invisible evil, even if the price to pay was their isolation. But while temporary isolation can be well tolerated, long-term isolation leads to a range of affected health outcomes and a high risk of early death for the elderly. Four months after they were forced into total isolation (first wave of the COVID-19 pandemic), the harmful impact of that isolation is now visible. Without any face-to-face relationships with their families, a reduced social network and loneliness have led insidiously to generalized anxiety and depression disorders in the very people we were trying to protect (4–8). In this regard, Grossman et al. studied the link between the loneliness-sleep problems of 243 Israeli older adults (mean age = 69.76, age range = 60–92) and their COVID-19 related worries and psychosocial resilience (9). These authors observed that this pandemic impacted psychosocial well-being and the rate of sleep problems was associated with COVID-19 related worries, and was inversely related to resilience (9). Their study then confirmed the relationship between loneliness and sleep problems observed in a large series of non-COVID-19 older patients in the UK (10). In addition, they suggested that the elderly may not represent an homogeneous population and were not equally affected by the restrictions imposed during this pandemic. A major parameter may be the access or literacy to digital resources that allow the elderly to be more socially connected, at least virtually, and to reduce the perception of isolation. Using evidence-based practice can inform decision-making. In this context, specific studies are therefore mandatory to explore the risk and protective factors for improving psychosocial health and the well-being in elderly.

The present opinion paper is focused on older adults living in care homes. However, care homes are not available or accessible in all countries. We need to explore how much the modalities of care have an impact on emotional isolation or abandonment of the elderly during the COVID-19 crisis. A recent Japanese study gave evidence of the deleterious effect of COVID-19 in older adults (11). Japanese society is considered to be more protective for older adults that many Western countries as shown by the higher life expectancy of Japanese elderly that can be explained by socio-cultural differences (e.g., family care, nutrition) and health system organization (12). Yamada et al. investigated changes in physical activity of 1,600 older adults living in community-dwelling before the COVID-19 pandemic (January 2020) and during the public health crisis (April 2020) (11). Despite the favorable organization of Japan for older adults, they reported a significant decrease in physical activity in these individuals that may lead to a higher risk of frailty and disability. Elderly who have restricted mobility and social connections may higher odds of facing adverse health outcomes, now and in a near future. It is urgent to evaluate whether the lifestyle of elderly (e.g., care homes or communities) can impact biopsychosocial outcomes amid this pandemic. Such studies should help to improve the psychological support of older adults during mobility restriction and lockdown.

In nursing homes, the protectors have gradually, and against their will, become jailers responsible for vulnerable residents who asked for nothing and have been deprived of their freedom. No visits and an obligation to stay in their rooms with the television as their only external contact are the rewards our elders have been given, with the sole aim of protecting them. As the lockdown measures seemed to have worked in many places, governments have relaxed, but visits from families remain under control, and limited in both time and number, rather like prison visits, but always for the good of our elders. No one has taken responsibility for releasing the elderly so that they can finally see their families, with suitable sanitary conditions. Care home staffs are waiting for directives which are slow to arrive and have to welcome families in drop-by appointments as if they were going to the hairdresser. Everything is under control, but most of all their freedom. Families are helpless in the face of these professionals who are certainly doing their best, but are in roughly the same psychological state and who are afraid of reprisals should contamination occur. The budget is constant or reduced but related to an increase. The hygiene rules, decided by the very highest authorities of the State, are implemented, even if they are sometimes/often not applicable.

Research lines are urgently needed to define specific recommendations related to reopening care facilities and services. Tele-health may be an interesting option for improving connectedness and minimizing the risk of infection exposure. Goodman-Casanova et al. conducted a survey to explore the impact of lockdown on the health and well-being of community-dwelling older adult (13) and to evaluate the benefit of television-based and telephone-based health and social support. These authors reported that the physical and mental health and well-being was worse during COVID-19 confinement and interestingly television-based health programs may offer potential benefits improving cognitive stimulation (e.g., recreational activity, memory exercises as intellectual activity) among this vulnerable population in addition to improve the medical follow-up. Television was the technological devises preferred by the older adults. Such approaches may help the practitioners and policymakers to protect the elderly during and after the COVID-19 crisis and the future pandemics (14).

## Conclusion

These rules have reduced most care home staff to the role of benevolent prison guards. People die slowly in total indifference, leaving their families in disarray. Single thinking, “good thinking,” has won; our responsibility was to protect the old, so we isolated them. Some countries allow election and music festivals when the elderly should stay cloister in their chambers to limit the propagation of the virus. There is something strange, isn't it? No one wants to take responsibility, but if a resident were to be infected, who would actually be responsible? Who has taken any interest in the wishes of the elderly, whose voice is just as respectable and just as important as that of the young? The desire to do good at all costs has led to what we are seeing today: the elderly remain isolated, to the indifference of everyone, and any words spoken in favor of their freedom remain unheard. Who is responsible? The politicians who decide, the scientists who advise, the medical staff who obey, or our societies who judge and blindly impose their rules? The economy is gradually starting to recover, borders are reopening, yet the elderly are still locked up and forgotten. In this context, the future for our elders is bleak and COVID 19 seems to have sealed their fate. If governments and companies do not rapidly take measures to reopen care home services and allow the elderly visits from their families with the appropriate means of protection, their deaths will be announced and this time, COVID 19 will not be directly responsible. Common sense must prevail, and we alone must be responsible for it.

## Author Contributions

The author confirms being the sole contributor of this work and has approved it for publication.

## Conflict of Interest

The author declares that the research was conducted in the absence of any commercial or financial relationships that could be construed as a potential conflict of interest.
